# Issues and principles for legitimate and meaningful One Health monitoring

**DOI:** 10.3389/ijph.2026.1609612

**Published:** 2026-05-18

**Authors:** Juliette Colinas, Manon Boiteux, Cécile Aenishaenslin

**Affiliations:** University of Montreal, Montréal, QC, Canada

**Keywords:** composite indicators, context-sensitive measurement, legitimacy, methodological principles, systems thinking

## Abstract

Although the One Health (OH) approach has gained prominence in health governance, no widely accepted method exists to assess the overall state of health across these interconnected domains in geographically defined settings. Available data remain fragmented, and existing evaluation frameworks largely focus on OH implementation rather than its outcomes. Two composite indices—the One Health Index and the Global One Health Index—have attempted to operationalize OH measurement, but their high level of aggregation, limited transparency, and top-down design constrain interpretability and legitimacy. We outline conceptual, logistical, and political challenges associated with measuring and monitoring OH outcomes, examine existing indices to illustrate these limitations, and argue that progress in OH measurement relies less on developing a single universal index than on establishing shared methodological principles. We propose core principles grounded in transparency and contextual relevance and highlight the need for collaborative efforts to develop clearer methodological guidance for more legitimate and meaningful OH monitoring systems.

## Introduction

Recent crises such as the COVID-19 pandemic and the continued spread of antimicrobial resistance (AMR) have revealed how deeply the health of humans, animals, and their environment are interconnected. They also exposed the institutional and interdisciplinary gaps that hinder coordinated responses. In this context, the One Health (OH) approach, defined by the Quadripartite Alliance as an integrated approach to sustainably balance and optimize the health of people, animals, plants and ecosystems, has become a guiding concept of health governance [1]. As part of its One Health Joint Plan of Action, the Quadripartite Alliance called on member states to develop and implement their national action plans for OH, and several have done so. But despite these advances, there is still no agreed method to assess and monitor over time the overall state of health across the human, animal, and environmental domains within geographically defined settings [1, 2].

The need for developing indicators to monitor OH across geographical settings and scales has been raised [1], as most evaluation tools for OH have focused so far on evaluating progress in the implementation of the approach, rather than on its health outcomes, such as the “One Health-ness” metrics developed by the Network for Evaluation of One Health (NEOH) [3–7].

The attraction of an integrated OH index is clear: it could help track progress over time and raise political visibility. Yet, experience from other domains such as sustainability science, development economics, and public health, shows that indices are rife with challenges and far from neutral: they involve methodological choices, hide trade-offs, and can unintentionally shape priorities [8–10].

In a context where there is strong demand for OH metrics, the objective of this article is to discuss major challenges associated with the development and use of indices for this field. We first outline the conceptual, logistical, and political challenges that complicate OH measurement, then characterize existing indicators, and finally propose principles for more legitimate and useful OH monitoring.

## Conceptual, logistical, and political challenges associated with measuring One Health

An indicator is “a quantitative or a qualitative measure derived from a series of observed facts” [9] that can be used to evaluate the state of some entity. More complex entities can be evaluated with several indicators to form a “composite indicator”. This set of indicator values can then be further manipulated through aggregation, i.e., some mathematical manipulation such as averaging, to obtain a new, single value, called an “index”. In this section, we highlight three levels of challenges linked to the development and use of such an index for measuring progress in One Health in geographically defined settings.

### Conceptual challenges

Developing an OH index inevitably involves simplification of a concept that is complex and multidimensional. According to the Quadripartite Alliance’s definition, developing an OH index requires first identifying metrics capable of assessing the state of health of humans, animals (domestic and wild), plants, and ecosystems, and then assessing the balance between these “healths”. Yet, how we perceive and define the health of humans, animals, plants and ecosystems, and more specifically, what constitutes a good balance between these “healths”, depends on diverse contexts, values, and worldviews. Choosing how to measure these “healths” and their relationships is therefore not straightforward, and attempting to apply this choice uniformly across all contexts risks overlooking local realities, priorities, and/or ethical positions [2, 11].

Once a working conceptualization of human, animal, and ecosystem health is in place, measuring them can take different forms. Metrics must be chosen to evaluate specific aspects of each domain. To produce the final values, the chosen metrics may then be combined partially to form a composite indicator, or entirely to form a single overall index. Each step in this process involves successive choices about how to group metrics, how to weigh them, and how to aggregate them; and each step entails a degree of simplification and a corresponding value judgment [9].

Aggregation itself is particularly tricky. On the one hand, when applied within a well-defined domain, it can help combine several indicators to approximate properties that are not directly observable through single measures, such as certain aspects of ecosystem health. In this sense, limited aggregation can support the measurement of complex concepts. Aggregation also makes it easier to communicate trends over time, as it reduces complexity to a single measure. On the other hand, aggregation reduces transparency and may obscure underlying dynamics, especially when applied across heterogeneous or non-substitutable components. In addition, the higher the level of aggregation, the more an index hides internal trade-offs because gains in one area can coincide with losses in another. In systems where trade-offs are an intrinsic feature, such as for OH, they are bound to occur. For instance, in certain settings, ecological conditions that foster biodiversity may also increase exposure to vectors and pathogens [12].

These tensions do not make measurement impossible, but highlight that indicators must be grounded in theory yet flexible enough to reflect context-specific meanings of health and interdependence. In practice, this requires transparency about the assumptions embedded in any evaluation framework, and openness to plural interpretations of what constitutes balance or resilience in OH systems.

Such flexibility is also essential because OH systems are dynamic: ecological conditions, health priorities, institutional capacities, and socio-economic constraints may evolve over time. Monitoring frameworks must therefore remain open not only to contextual variation, but also to changes in how results are interpreted and used for decision-making, as well as to evolving knowledge that is still particularly limited in some OH domains.

### Logistical challenges

Indicator choice is also contingent upon logistical constraints that remain significant. Reliable and comparable data are unevenly distributed across OH domains: animal and ecosystem data are often sparse or non-standardized, and even human-health data differ in quality and coverage across regions. These disparities limit the feasibility of indices defined at broad geographical scales and point to the need for tiered approaches that build from what is locally measurable. In addition, technical issues such as data access, interoperability, and validation, are compounded by resource asymmetries and differing national priorities. In some settings, investing in long-term monitoring may compete with more immediate health, security and development needs [1, 13]. Strengthening data ecosystems, fostering open-data standards, and supporting regional networks are therefore important preconditions for credible OH measurement. Addressing these logistical constraints is not only a technical task but also a political one, as it requires sustained collaboration and equitable resource sharing across sectors and countries.

### Political and institutional challenges

Finally, decisions about what to measure, how to aggregate, and how to interpret results are inherently political, reflecting institutional priorities and implicit value judgements. When a small group of experts defines metrics for global comparison, or when funding is tied to index rankings, issues of legitimacy and accountability arise. Participatory and transparent design processes can mitigate these risks, but they require time, facilitation, and a sustained commitment to shared learning across sectors. It is therefore crucial to recognize that indicators can serve very different purposes—some oriented toward control and accountability, others toward dialogue and improvement. The latter orientation is more consistent with the spirit of OH, but needs to be considered before and throughout the entire development process, rather than only after the release of the indicator or index [8, 14].

## Currently available One Health indices (OHI and GOHI)

To identify published One Health (OH) indices relevant to our research question, we conducted a structured search in PubMed and Web of Science (February 2023, updated in November 2025) using combinations of the terms *One Health*, *Planetary Health*, *human*, *animal*, and *ecosystem health*, together with *index* and *indicator*. We also reviewed the SDG indicator framework, but no index meeting the OH inclusion criteria was identified. The search yielded 128 records after duplicate removal, which were screened manually and independently by two authors. Articles were eligible if they described the development or application of an index explicitly addressing the three main OH domains: human, animal, and environment/ecosystem health.

Two composite indices met these criteria: the One Health Index (OHI) and the Global One Health Index (GOHI). We reviewed and analyzed these two indices in light of the issues raised above and concluded that, although both represent significant progress in the monitoring of OH over time and space, they illustrate why assessing OH remains challenging in practice.

The OHI, developed for measuring OH in metropolitan areas in Brazil [15], uses 28 indicators derived from municipal datasets. These indicators are grouped in three aggregated scores: one for animal health, one for the health of the environment, and one for human health. However, most of the included indicators represent determinants of health in these sectors rather than health outcomes. The only outcome indicator is mortality, which is used for human health. Other examples of indicators include the presence of an animal protection plan and microchip identification (animal health), a city garbage plan and individuals with hoarding behavior (environmental health), and income and school attendance rates (human health). These indicators appear to be treated as determinants of health, but the mechanisms through which they are expected to affect OH outcomes are not clearly explained. Thus, while this context-specific design grounds the index in local realities, its framework is loosely anchored in OH theory. Some indicators appear driven more by data availability than conceptual relevance or established evidence, and several heterogeneous variables within each domain are aggregated into a single composite score. The resulting index combines heterogeneous dimensions related to sanitary conditions, environmental management and policy efforts, but its high level of internal aggregation and the inclusion of indicators whose relevance to OH outcomes is unclear limit its interpretability and usefulness for guiding integrated OH decision-making.

The GOHI [16] adopts a global perspective, integrating 57 indicators extracted from international databases to assess OH at the national level. It produces a single final aggregated score composed of three sub-indices called “external drivers,” “intrinsic drivers,” and “core OH capacities”. Each of these sub-indices combines highly heterogeneous indicators into single scores. For example, the intrinsic drivers sub-index includes indicators for cardiovascular disease, discarded fish, household solid fuels, fertilizer consumption, transparency, and risk communication. Thus, although the ambition to achieve global comparability is commendable, highly heterogeneous variables are aggregated within the same framework without clear justification. In addition, methodological transparency remains limited regarding indicator selection, weighting, and aggregation, which undermines the index’s “practical usefulness”, as underlined in a report for the European Commission [17]. Although the GOHI includes more health outcomes indicators than the OHI, it also merges broad categories of determinants (such as governance and socioeconomic drivers) with outcomes (such as disease burden) across and within domains, allowing strong performance in one area to compensate for weaknesses in another. Overall, this high level of aggregation masks tensions and trade-offs that are central to OH systems, while the exclusive reliance on quantitative data leaves little room for contextual or qualitative insights.

Importantly, both indices appear to have been primarily developed by experts (as described in the GOHI’s Methods and judging from the OHI authors’ affiliations). This brings us back to the question of legitimacy and accountability discussed above. Recent critiques of the GOHI also reflect these concerns regarding the design and use of such indices [17].

Overall, these two indices demonstrate both the promise and the pitfalls of current OH measurement efforts. They underscore that while composite indices can initiate dialogue and raise awareness, excessive aggregation, limited transparency, and top-down design reduce their interpretability and legitimacy. Their experience therefore reflects the conceptual challenges (how to define and represent health across domains in a meaningful and transparent way), logistical challenges (uneven data), and political challenges (limited participation and accountability) outlined previously. Together, these issues point to the need for a more flexible, conceptually grounded, and transparent approach—one that values contextual relevance over uniformity and that builds on methodological guidance rather than a unique measurement system.

## A way forward

The difficulty of developing meaningful OH indicators reflects what makes this approach distinctive: its commitment to relationships that cut across disciplines, species, and scales of governance. Measuring the evolution of such a system requires methods that respect this complexity rather than reduce it. Building on the observations made above, we identify a set of core principles to support the development and use of context-specific OH indicators. These principles are not intended as a complete implementation framework, but as a basis for broader guidance on indicator development adapted to different contexts.

### Clarifying purpose and design choices

Although we agree that composite indicators can play a valuable role in measuring OH outcomes, their design and purpose must be made explicit to prevent misuse and optimize their usefulness for decision-making at different levels. Lessons from other domains, such as sustainability science and the circular economy [18], show that progress depends less on producing ever-larger datasets than on clarifying how and why indicators are designed. Reflection on purpose is crucial: what is the indicator meant to reveal, and for whom? Throughout the process, designers should revisit basic questions—what to measure, how concepts are defined, and how measurability is balanced with relevance [18]. In this perspective, reliance on quantitative indicators alone is insufficient to capture the complexity and context-dependence of OH systems. Complementing quantitative data with qualitative or experiential insights can help make explicit assumptions, values, and system dynamics that would otherwise remain invisible.

### Accounting for multiple scales

OH monitoring should also account for the fact that health challenges unfold across multiple scales. Some issues require harmonized indicators and coordinated surveillance across countries, while others are more closely tied to local ecological, social, or institutional conditions and therefore call for context-specific approaches. A meaningful OH monitoring system should therefore be able to combine different levels of observation, rather than impose a single measurement logic across all scales and purposes.

### Minimal aggregation

In the context of OH indicators, we argue that aggregation should be limited to reducing redundancy within categories and avoided between non-substitutable components such as human, animal, and ecosystem health [19]. Moreover, a non-aggregated composite indicator, with several sub-indicators within each domain, could serve several purposes for which further aggregation is not only unnecessary but detrimental: identifying system interactions, tracing policy impacts across domains, informing policymakers on how to adopt a more holistic perspective of human, animal, and ecosystem health and their interconnections, and helping actors visualize complexity without erasing it [20–22]. Visualization tools—maps, color scales, radar charts—can facilitate interpretation without collapsing dimensions [20].

In addition, OH monitoring systems should make explicit whether they aim to assess health outcomes, health determinants, or both, and these dimensions should not be conflated without clear justification, as their relationships are often context-dependent and may vary across geographical, socio-economic, and institutional settings [23, 24]. Aggregating determinants and outcomes into a single score can obscure causal pathways and complicate interpretation. Greater clarity on these distinctions would improve transparency, support more meaningful comparisons, and better inform decision-making.

### Distributed capacity, shared responsibility

Together, the challenges reported in the previous sections suggest that a single top-down OH index is neither desirable nor feasible. Rather than seeking standardization through a single metric, progress depends on the development of shared methodological principles that can support legitimate and meaningful OH monitoring across diverse contexts. Such progress also requires methodological transparency, distributed capacity, and shared responsibility for interpretation.

The principles outlined in this paper are intended as a starting point. They are not meant to constitute a complete operational framework, but rather to highlight key methodological issues that should guide future work. More detailed methodological guidance will be needed to support the development of context-sensitive OH indicators across different settings and scales.

Such guidance could support reflection and decision-making on issues such as: Who should be included in the design process and, if participatory, which methods are appropriate? How can meaningful and balanced indicators of the One Health dimensions be selected? Should variability across space and socio-economic groups be assessed, and if so how? Why and how might qualitative data, system-level indicators, and indicators for different geographical scales be included?

A first step could be to establish an inclusive international working group to support the development of guidance for designing and implementing OH monitoring systems at different scales (local, national, international). Rather than prescribing a single model, such a group could provide shared principles, context-sensitive options, and advisory support to help local actors develop monitoring systems suited to their needs. The shared principles could include recommendations on key development steps, conceptual and methodological frameworks, stakeholders to be involved in the design, potential indicators to be included, data sources to be considered, options for data aggregation when suitable, and ways of interpreting outputs and linking them to decision-making. To strengthen legitimacy and contextual relevance, this group should not be composed solely of technical experts, but should also include a balanced representation from the main OH domains, policymakers from relevant sectors, and civil society representatives.

The shift in focus from top-down standardization to shared examination and methodological principles reframes measurement itself. It turns OH monitoring into a shared practice of observation—one that values transparency, contextual relevance, and mutual learning, as emphasized in sustainability and systems research [14, 18, 21]. Such an approach can help translate the OH vision into operational terms without compromising the complexity and diversity that gives it meaning.

### Box A. Policy implications

- Shift from prescription to guidance: International OH institutions and initiatives (such as the Quadripartite Alliance and related OH networks) should promote shared examination of existing indicators and the development of guidance for OH monitoring systems, instead of developing a single OH index.
- Recognize indicators as learning instruments: Use measurement to reveal trade-offs and support adaptive governance, not only accountability.

### Box B. Design principles

- Transparency and participation: Make design choices explicit and involve a diversity of stakeholders throughout the process to strengthen legitimacy and trust through a transparent participatory process.
- Integration of quantitative and qualitative insights: Combine measurable data with qualitative or experiential evidence to capture complex and context-dependent aspects of OH systems.
- Restraint in aggregation: Avoid combining non-substitutable domains; use visual or interactive tools (dashboards, radar charts, maps) to communicate multidimensionality without oversimplification.
- Iterative learning: Treat indicator development as a dynamic, reflexive process that evolves with scientific understanding and policy needs.

### Conclusion

Measuring OH is ultimately a question of how we choose to define, evaluate, and represent what we consider a healthy balance among human, animal, and ecosystem domains. These judgments of value cannot rest with a small circle of experts, nor can such interdependence be reduced to numbers that strip it of meaning. What is needed are OH monitoring systems capable of making the complexities of human, animal, and ecosystem health both legible and actionable. This requires moving beyond aggregated indices that oversimplify, toward measures that provide information, encourage reflection, and sustain collaboration across contexts and scales.
